# Disparities in breast cancer care and research: report from a Breast Cancer Research Foundation sponsored workshop, 9–10 October 2014

**DOI:** 10.1038/npjbcancer.2015.13

**Published:** 2015-10-14

**Authors:** Beverly Canin, Karen M Freund, Patricia A Ganz, Dawn L Hershman, Electra D Paskett

**Affiliations:** 1 Breast Cancer Advocate, New York, NY, USA; 2 Tufts Medical Center and Tufts University School of Medicine, Boston, MA, USA; 3 UCLA Schools of Medicine and Public Health, Los Angeles, CA, USA; 4 Center for Cancer Prevention and Control Research, Jonsson Comprehensive Cancer Center, University of California, Los Angeles, Los Angeles, CA, USA; 5 Columbia University, New York, NY, USA; 6 Ohio State University, Columbus, OH, USA

## Abstract

The purpose of this workshop was to bring together diverse stakeholders from the breast cancer research community to discuss critical issues related to disparities in breast cancer care and to identify potential strategies for reducing disparities and inequities in care through research. The workshop format included a series of formal content presentations, participation in break out groups that focused on specific topics highlighted in the content presentations, reporting back of findings and a facilitated discussion that focused on shaping a research agenda. The workshop members concluded that numerous groups of women are at increased risk for disparities in breast cancer care: many patients and survivors suffer disproportionately from inadequate access to high-quality diagnosis and treatment, resulting in more frequent and severe adverse outcomes from the disease. Research on breast cancer disparities provides a major opportunity for reducing the burden of breast cancer. Thus, it is important for the Breast Cancer Research Foundation and other research funders to consider how to best promote research focused on ensuring breast cancer health equity.

## Introduction and purpose of workshop

**Figure 1:**
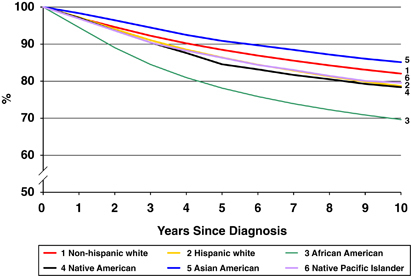
Female breast cancer survival by race/ethnicity, from SEER data, 1995–2010.

**Figure 2:**
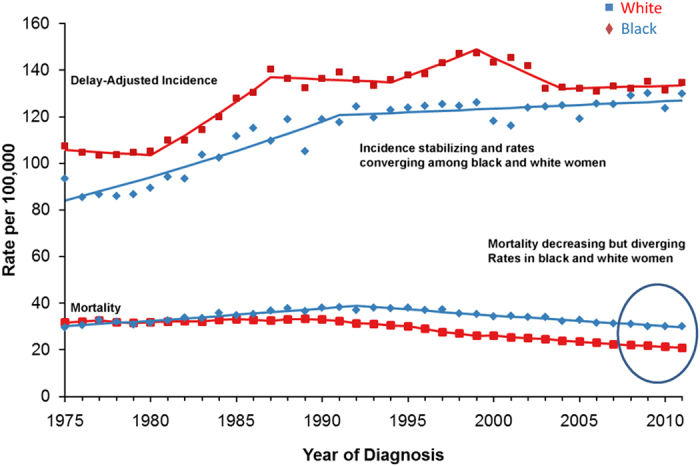
Incidence and mortality data from SEER 9 delay-adjusted rates 1975–2011.

At the January 2014 meeting of the Breast Cancer Research Foundation (BCRF) scientific advisory board, members noted that disparities in breast cancer care and outcomes were a growing concern (Figures 1 and 2). Breast cancer research focused on decreasing risk, reducing morbidity, and increasing cures is the mission of BCRF—but must include a focus on disparities (identification of issues and solutions) to be fully comprehensive and effective. As a result, a working group of BCRF investigators was convened to discuss this issue and think about strategies to identify relevant research opportunities. Among the topics considered for discussion were the widening gaps in survival, even while there has been extraordinary progress in basic, translational, and clinical research yielding more effective new treatments. To address these concerns, a subsequent workshop was held, bringing together researchers, clinicians, public policy professionals, community leaders, and patient advocates, to identify opportunities and challenges in the conduct of research focused on disparities in breast cancer care and outcomes. This report is a summary of that workshop.

## Keynote address

**Figure 3:**
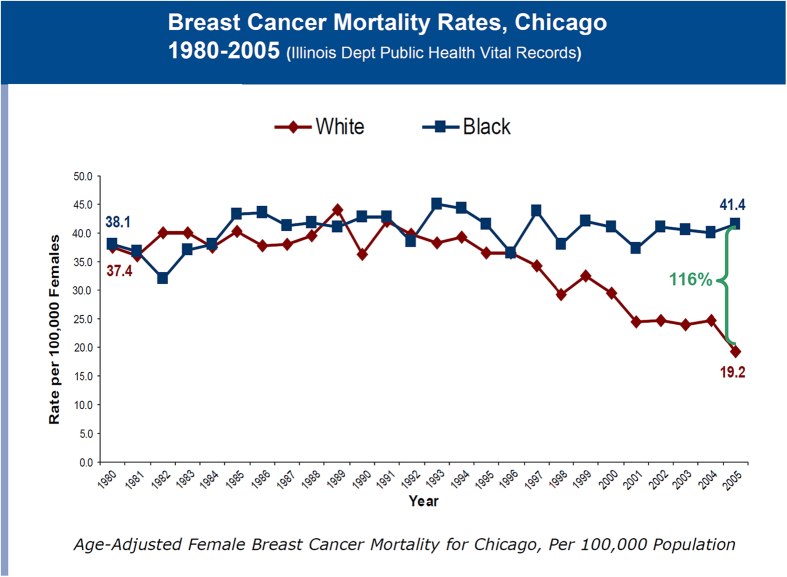
Age-adjusted breast cancer mortality rates in Chicago, adapted from ref. 1.

Dr Carol Ferrans, of the University of Illinois at Chicago, described an effort by multiple stakeholders in Chicago to address the worsening gap in survival outcomes between black women and white women with breast cancer.^1^ From 1980 until 1996, Chicago mortality rates for black and white women with breast cancer were about the same, but by the late 1990s a widening gap was emerging. By 2005, the mortality rate for black women was 116% higher than the rate for white women. (Figure 3) This alarming information galvanized community stakeholders from health care, public health, advocacy, and the government to form the Metropolitan Chicago Breast Cancer Task Force. Dr Ferrans explained the process of building the local task force, identifying the needs and forming recommendations that could have a measureable impact on breast cancer disparities. She shared successes and challenges in implementing recommendations, such as lack of financial resources to support program recommendations, lack of access to quality mammography, and cultural barriers to screening and treatment. She emphasized the importance of prior research to show a compelling need (shining the light on the problem making it impossible to ignore), of making the community and legislature aware of the need through widespread media coverage and advocate involvement, and of creating incentives for health-care providers to participate. Major outcomes of the effort were passage of the Illinois Reducing Breast Cancer Disparities Act, creation of the Breast Cancer Quality Consortium to improve mammography quality and expansion of the Illinois Breast and Cervical Cancer Program to cover all uninsured women in Illinois, for screening, diagnosis, and treatment.

## Summary of core presentations

### Cross-cutting themes and special populations

Providing an overview of themes and challenges in addressing disparities in ethnic minority and underserved communities, Dr Roshan Bastani, of the Fielding School of Public Health at UCLA, began with commonly understood definitions of health disparities and health inequities. A health disparity is generally interpreted as a difference in the presence of a disease, in health outcomes, or in access to care among population groups. The term health inequity is increasingly being applied to describe differences or disparities in health status and health outcomes that are unnecessary, avoidable, and also considered unfair and unjust. Therefore, health equity is the absence of unfair and avoidable or remediable differences in health outcomes. Healthy People 2020 defines health equity as attainment of the highest level of health for all people (http://www.healthypeople.gov/2020/about/foundation-health-measures/Disparities). Dr Bastani focused on the current framework of disparities research and the limitations in how disparities research is funded and conducted, which impede a true understanding of community need and impacts on the point of service, care, and health-care outcomes. In order to achieve health equity, Bastani suggested that the language and culture of disparities research must change. Disparities research must be conducted in a community-based participatory model involving transdisciplinary teams (representatives from basic, clinical, psychosocial sciences, public health, and policy) and representation from the community. It must first take into consideration community needs and priorities. Also, she said, identifying similarities in disparities across populations and devising solutions that apply to multiple disadvantaged groups is more sustainable and thus more likely to yield population-wide impact.

### Special issues in urban, poor, and minority communities

Dr Nina Bickell, from the Mount Sinai Center for Health Equity and Community Engaged Research, highlighted access to high-quality cancer care and health insurance as the predominant drivers of disparities in breast cancer care and outcomes in poor urban communities. Important factors that can improve clinical care in low-resource settings are information sharing among the clinical care team, tracking of follow-up, patient-centered culture, adequate systems support (organizational and administrative) at the point of care, and flexibility of the health-care workforce to work around system limitations. Challenges include ensuring access to advanced cancer care, such as palliative and end-of-life care, and access to clinical trials.

### Disparities in breast cancer care in rural communities

BCRF investigator, Dr Electra Paskett, from the Ohio State University Comprehensive Cancer Center, cited certain statistics related to rural populations: 72% of the geographical US is rural; 15% of the population resides in rural areas; 2.8 million cancer survivors live in rural areas of the US. She noted that rural communities are diverse in both geography and racial/ethnic populations. These communities are understudied and face a variety of disparities in health care due to geographical isolation and barriers imposed by cultural beliefs, lack of education, lower economic status, and co-morbidities. Rural women with breast cancer face barriers at all points of the cancer care continuum from screening to survivorship and end-of-life care are less likely to receive standard of care or have adequate psychosocial support, and are more likely to suffer from depression and have poorer quality of life after breast cancer. Treatment decisions are often made based on transportation and accessibility rather than medical need. Tailored interventions are needed for this population to improve access to screening and prevention counseling and to improve the patient’s experience with treatment, follow-up, and survivorship.

### Disparities and challenges in meeting the psychosocial needs of patients with breast cancer

BCRF investigator, Dr Annette Stanton, from the Department of Psychology at UCLA, noted that disparities in the impact and effective management of psychosocial concerns in breast cancer patients are not well studied and that much more work needs to be carried out. Research regarding the intersecting influences of ethnicity/race, socioeconomic status, and cancer variables (e.g., disease stage) on psychosocial outcomes is needed. Disparities in psychosocial outcomes exist between Latina American breast cancer survivors and non-Latina counterparts independent of economic status.^2,3^ Understanding of the contributors to such disparities, as well as the determinants of positive psychosocial outcomes, is necessary to inform targeted and culturally tailored interventions.

### Disparities in care experienced by older patients with breast cancer

BCRF investigator, Dr Arti Hurria, from the City of Hope Cancer Center, noted that older patients have poorer outcomes than younger patients for a variety of reasons. Older patients are a very heterogeneous population regarding overall health and physiological status even before being diagnosed with breast cancer. They are rarely included in clinical trials, often due to co-morbidities; consequently, many breast cancer therapies have not been adequately studied in this group. Efforts to identify challenges and opportunities in geriatric oncology are ongoing as part of a collaborative geriatric oncology consortium Dr Hurria is leading. She presented a model for clinical trials in both the metastatic and adjuvant settings to test cancer drugs in older patients in order to better understand their side effects and to expand data collection to capture the characteristics of this population. This is a serious need as the advances made in the laboratory and clinic cannot be extended to older patients with breast cancer until they are adequately evaluated in this target population.

### Synopsis of findings from a collaborative think tank on health disparities

Dr Blase Polite, from the University of Chicago, gave the final presentation, which was a synopsis of findings from a meeting attended by representatives from the American Society of Clinical Oncology, the American Association for Cancer Research, the National Cancer Institute and the American Cancer Society in February 2014. The goals and objectives of that meeting were to discuss the state of the science on health disparities to inform a joint statement from these organizations which would make key recommendations for research priorities and for improving how disparities research is conducted and disseminated. Dr Polite described the specific areas that were discussed including many important research areas that need further development and implementation. A report from that effort will be forthcoming, and should be very valuable to the research community in moving the field forward. Many of the topics identified for discussion in this workshop overlap with those discussed by those who attended the think tank described by Dr Polite.

## Work group discussions

### Community-based research strategies

This work group identified several strategies for addressing breast cancer disparities. Although a Community-Based Participatory Research (CPBR) strategy is optimal for assuring community buy-in and having a sustainable intervention, accomplishing full participation is not always possible. A more feasible approach is to use a multilevel strategy that involves building relationships in the community and creating infrastructure to conduct research that addresses community needs, especially in the populations that suffer disparities. Policy makers need to be convinced that addressing disparities is a good investment—economically as well as from the human perspective. Systems and structures that can help deliver and sustain the interventions, e.g., federally qualified health centers, health departments, etc., must also be targeted, before following with interventions for individual patients (e.g., assess risk and screening). Outcome aims for the reduction of disparities cannot be limited to mortality due to time needed to accrue results. Other important outcome aims include use of mammography, time to notification of abnormal test results, and time to resolution/diagnosis/treatment. Although using CPBR is ideal, it takes time to build trust within the community, especially as certain elements inherent within research often create delays and problems—e.g., IRB’s, control groups, and consent forms. One way to assist with building trust and gaining entrée into communities to address disparities is to use trusted community members such as community health workers or lay patient navigators.

### Disparities in clinical care and clinical trials

Five areas were identified where clinical care provision and clinical trial design could have a major impact in reducing breast cancer disparities.

#### (1) Guideline concordant care

Data from a number of sources suggest that women from minority communities are less likely to receive guideline recommended care.^4–6^ The group identified the need for monitoring of specific quality of care metrics in breast cancer care. Monitoring serves to specifically identify populations, locations, and processes of care in need of intervention as well as to provide the impetus for intervention. Consensus development of a group of metrics that balance feasibility and utility to address gaps in care is needed.

#### (2) Eligibility expansion to include those with common medical co-morbidities

This strategy builds upon strategies for inclusion of elderly into clinical trials.^7^ Co-morbidities are one of the major reasons of ineligibility for clinical trials by minority groups^8^ and the lack of diversity in clinical trial populations. Lower income is also a barrier.^9^ To more rapidly expand data on the effectiveness of new therapies to underserved populations, processes of concurrently expanding cohort eligibility, or enrichment or extended trial design for common stable co-morbidities (including diabetes, hypertension, coronary artery disease, and chronic renal disease), could rapidly address disparities and facilitate more rapid dissemination of findings to a broader patient spectrum.

#### (3) Simplification of therapeutic protocols

The increased complexity of clinical cancer care results in lower adherence among those with low health literacy and other barriers including insurance instability, housing, and income instability, all of which put completion of therapy at risk. Explicit National Institutes of Health and funder goals of reduced complexity in new protocols may serve to reduce disparities in treatment adherence.

#### (4) Coordination of care between health-care providers

Bickell’s findings^10,11^ and the growing literature within and outside of cancer have demonstrated quality gaps that accompany the increasingly more complex care, whether inpatient or outpatient care, primary, or specialty care.^12^ Evaluation and dissemination of models of coordination are needed. Stanton’s findings^13,14^ and others^15^ of the large unmet psychosocial needs of underserved populations speaks to the explicit need for these coordinated models to include behavioral health.

#### (5) Implementation and dissemination

Implementation research is needed to determine best methods to disseminate known effective strategies to improve quality of care, such as patient navigation.^16^ Broad stakeholder engagement is needed to develop these five targeted areas of improvement in clinical trials enrollment among minority and underserved populations, and for improved coordination and quality of clinical care. For example, the pharmaceutical industry along with the Food and Drug Administration and cancer trial groups need to jointly develop systems to promote protocol simplification and expanded cohort eligibility. Providers, accountable care organizations, and insurers are currently well motivated to work together to implement care coordination as well as quality metrics and methods to adopt interventions shown to address disparities.

### Breast cancer and aging

Significant age-related disparities in breast cancer care and outcomes were reviewed. From 1990 to 2007, breast cancer death rates decreased by 2.5% per year in women age 20–49 years, but only 1.1% per year in women age ⩾75 years.^17^ As breast cancer is a disease associated with aging and the number of older adults with breast cancer is expected to greatly increase over the next decades, this age-related disparity in breast cancer outcomes is of great concern. Five targets were identified to improve evidence-based research in older adults, with the goal of subsequently decreasing disparities.

#### (1) Improve evidence-based research in older adults with breast cancer

In particular, there is a need to bolster the enrollment of older adults in phase III studies, which define the standard of care. As older adults have been under-represented in breast cancer clinical trials, particularly adjuvant and Food and Drug Administration registration trials,^18^ a several-pronged approach for filling this knowledge gap is recommended, including recommendations from the Institute of Medicine^19^ and Cancer and Aging Research Group.^20^ Studies are needed to pinpoint the enrollment barriers for older adults and to find ways to overcome these barriers. Just as researchers design a plan that includes recruitment of women and minorities, there should be a similar plan to enroll enough older adults in trials for the age distribution on the study to mimic that of the disease. In pediatric medicine, a patent extension is employed if the drug is studied in children. A similar principle could apply to older adults, with policy makers and key stakeholders integrally involved in the effort.

#### (2) Prospectively collect functional status of older adults enrolling in trials

This includes measures of functional (as opposed to chronological) age, which is captured by items included in a geriatric assessment. This information could help researchers and clinicians look past chronological age to ‘functional age’ when tailoring a treatment plan to an individual. Also, tools are needed to facilitate decision-making and incorporate preferences of older adults in the care plan, taking into account both the short- and long-term side effects of cancer therapy.

#### (3) Understand how cancer and cancer therapy impact the health and well-being of older breast cancer survivors

With improvements in screening and therapy, the number of older cancer survivors is growing. Studies of the short- and long-term impact of cancer and cancer treatment on overall health, function, and cognition of older adults are needed in order to develop interventions to promote health and well-being after treatment.

#### (4) Facilitate physical access to care for older adults with breast cancer

Patients with limited mobility and/or social support will experience challenges traveling long distances to receive treatment. Factors contributing to patient and caregiver burden that may impact access to care must be considered. The direct and indirect cost of caregiving also needs to be accounted for.

#### (5) Engender cross-talk between geriatrics and oncology

The majority of patients with cancer are older adults, yet very few—if any—geriatric principles are included in oncology training. Furthermore, most geriatricians and primary care providers have limited education in oncology care. This represents an opportunity for transdisciplinary education to improve the quality of cancer care for older adults.

### Disparities in psychosocial services and survivorship care

Future research on disparities in psychosocial services and survivorship care needs to consider access and social–cultural barriers. One way to begin to address these factors is to collect data on psychosocial factors, e.g., depression, social isolation/loneliness, and fatalism. This would facilitate research being carried out with patients and could be associated with earlier clinical intervention when a problem is first identified; thus, adding to the cost-effectiveness of this strategy. Treatment modalities for psychosocial problems need further development. Things to consider when developing these modalities include who is and is not participating in treatment, allowing interventions to be tailored or adapted, defining protective factors, as well as risk factors, testing interventions with diverse populations, keeping in mind the need to maximize adherence to any intervention implemented, and involving the community in development. Additional questions apply to underserved populations when considering the development of treatment interventions—for example, is there an extra burden of psychosocial risk factors in underserved populations and in what diverse cultural contexts might these risk factors be more likely to occur causing increased risk for adverse events. Finally, to realize the benefits of effective interventions, patients need to be invited to participate, perhaps by discussion of the benefits of treatment to themselves, their families, and their communities.

## Shaping a research agenda: from cells to society

In the final session of the workshop, BCRF investigators facilitated a summary discussion that wove together themes and content that was heard across the various work groups, with a focus that extended from the biological to the societal. Biological differences in host factors can contribute to breast cancer disparities. Conditions associated with poorer cancer outcomes, including obesity, stress, and chronic inflammation, are highly prevalent in lower socioeconomic status populations. Their impact on development and progression of cancer are critical areas to elucidate. The interactions among biology, environment, and behavior along with their differential impact on cancer outcomes pose important areas to be further researched. The inevitable large amounts of personalized data from genetic testing, merged with clinical electronic medical record and environmental and administrative databases, offer tremendous opportunities to examine promoters of carcinogenesis and areas to intervene to reduce disparities in cancer outcomes.

In Table 1, we summarize the strategies that were identified in each domain that was considered. As can be seen, there is much to be done, across the continuum from the most basic biology to policy. A major theme that permeated the discussion was the importance of acknowledging the high societal cost of disparities in breast cancer care. If this is not a priority for all research scientists, it will not be effectively addressed.

## Conclusions

Breast cancer disparities are a major concern for many groups, including racial/ethnic minority, older, rural, less educated, the underserved, those with cultural barriers, and breast cancer survivors who have ongoing medical or psychosocial needs. Many cancer patients and survivors suffer disproportionately from inadequate access to high-quality diagnosis and treatment and more frequent and severe adverse outcomes from the disease. Basic and implementation science research and the application of evidence-based research results on breast cancer disparities are essential to achieve good health of at-risk communities and for our society as a whole. Future research will benefit from involvement of transdisciplinary teams including academic, public health, and community leaders, and delineating paths through which the research can be translated into wide-scale public benefit. Thus, it is important for BCRF and other funders of research to consider how they can best promote research that is focused on eliminating breast cancer health disparities.

## Workshop Attendees

Roshan Bastani, PhD, Fielding School of Public Health, University of California, Los Angeles, CA

Nina Bickell, MD, Mount Sinai Hospital, New York, NY

Beverly Canin, Breast Cancer Advocate, New York, NY

Graham A Colditz, MD, DrPH, FAFPHM, Washington University, St Louis, MO

Elise D Cook, MD, MS, University of Texas, MD Anderson Cancer Center, Houston, TX

Carol Estwing Ferrans, PhD, RN, FAAN, University of Illinois at Chicago, Chicago, IL

Karen M Freund, MD MPH, Tufts Medical Center and Tufts University School of Medicine, Boston, MA

Patricia A Ganz, MD, UCLA Schools of Medicine and Public Health, Los Angeles, CA

Sarah Gehlert, PhD, Washington University, St Louis, MO

Peter Greenwald, MD, DrPH, National Cancer Institute, National Institutes of Health, Bethesda, MD

Dawn L Hershman, MD, MS, Columbia University, New York, NY

Clifford Hudis, MD, Memorial Sloan Cancer Center, New York, NY

Arti Hurria, MD, City of Hope, Duarte, CA

Elena Martinez, PhD, University of California, San Diego, La Jolla, CA

Jewel Mullen, MD, MPH, MPA, Department of Public Health, CT

Electra D Paskett, PhD, MSPH, Ohio State University, Columbus, OH

Edith A Perez, MD, Mayo Clinic, Jacksonville, FL

Blase Polite, MD, University of Chicago Medicine, Chicago, IL

Julia H Rowland, PhD, National Cancer Institute, Cancer Control and Populations Sciences

Patricia Spears, Advocate, North Carolina

Annette L Stanton, PhD, University of California, Los Angeles, CA

Sandra M Swain, MD, MedStar Washington Hospital Center and Georgetown University, Washington, DC

Mary Beth Terry, PhD, Columbia University, Mailman School of Public Health, New York, NY

Beti Thompson, PhD, Fred Hutchinson Cancer Research Center, Seattle, WA

BCRF staff: Ms Margaret Mastrianni and Margaret Flowers, PhD

## Figures and Tables

**Table 1:** Cross-cutting research strategies to reduce breast cancer disparities

*Tumor biology and host factors*
Promote interdisciplinary research to expand the approach of the science of health disparities; e.g., the biology of obesity, an important risk factor that is prevalent in low-income populations.
Use clinical trial biospecimens for correlative studies that can examine host factor biology; e.g., chronic inflammation and its role in cancer progression.
Identify biomarkers and biological measures associated with socioeconomic disadvantage; e.g., cortisol levels as a measure of stress.
Develop a set of standardized tools for measurements of cancer disparities, i.e., questionnaire, biological and outcomes measures.
Integrate data on both biological and behavioral assessment of the individual.
Identify methods to assess the whole person with both qualitative measures and biological measures.
Develop strategies for the use of inevitably large amounts of data from personal genetic tests that will become more common in the next 5–10 years.

*Individual level: whole -person care*
Develop multidisciplinary assessment to address whole-person care
Include perspectives of primary care providers, other health-care providers.
Address family history.
Recognize cultural barriers in talking about breast cancer/breast health.
Acknowledge barriers due to maternal and caretaking roles; e.g., mother takes care of others first, her health comes second.
Must take a life course perspective, and address opportunities for prevention and intervention across childhood, adolescence, young adult, middle age, and older ages.
Develop primary and secondary prevention strategies
Learn from cardiovascular prevention experience.
Develop implementation strategies for evidence-based approaches to behavioral modifiers; e.g., exercise and diet.
Develop preventive strategies that address exposures beginning *in utero*.
Improve adherence to age appropriate mammographic screening.
Develop strategies for physician reinforcement of preventive recommendations.
Develop outcomes that address the whole person: treatment, follow ups
Include multidisciplinary care team so that psychosocial, behavior, and functional outcomes included.
Include outcomes of impact of cancer and care on members.

*Social/environmental context*
Understand the barriers to accessing the medical care system by vulnerable populations
Fear and trust issues.
Safety/stress issues; lack of access to safe environment.
Develop and assess community and environmental interventions.
Develop partnerships/alliances with local programs.
Advocate for legislation to reduce exposures, and policies to provide incentive and support for healthy lifestyle choices, incentivize healthy choices.
Develop workplace/church interventions/incentives, with the goal of changing the environment at work (in the community) leading to changes in behavior at home.
Develop implementation strategies for environmental exposures that move from the workplace to the community
Incentivize smaller companies with bottom line on investment in healthy lifestyles to improve productivity and reduce loss of work hours.

*Institutional considerations*
Develop a multi-factorial perspective, including the integrated attention of many disciplines both within and outside of the health sector. Transdisciplinary multilevel research is particularly important for addressing breast cancer disparities in communities.
Focus on the lack of or insufficient health-care coverage and low socioeconomic status (e.g., as measured by income, education level, and occupation) as one of the strongest factors in disparities. These factors influence the incidence, prevalence, mortality, and burden of the disease.
Evaluate all the resources needed to cover costs of breast cancer detection, treatment, and long-term care.
Measure contrasting rates across populations, such as poverty, education, and racial/ethnic patterns, for planning and evaluating intervention programs.
Value and support community engagement in institutional interventions. This is now a key consideration for research funding by foundations and agencies.
Build upon community assets when developing interventions, e.g., cultural competence, committed community leadership, coordinate, and build on preexisting organizational structure.
Develop specific interventions to identify and improve organizational and structural characteristics that contribute to cancer disparities in institutions serving primarily vulnerable populations is paramount.
Develop strategies that attend to education and knowledge of the patients and/or family members and their role as a resource during breast cancer detection, treatment, and long-term care.

*Policy issues*
Implementation research on methods to adopt of evidence-based recommendations.
Measure and report the impact of disparities on public health and the ongoing costs of inaction, formatted with the language of policy makers.
Change the language from disparities to ‘achieving health equities’, with a focus on positive aspects of achieving health equity in both health and economic terms.
Focus new initiatives on breast cancer risk reduction
Address women’s health/family life issues
Health promotion messaging.
Ethnic communities, cultural norms, and women’s role.
Identify similarities between rural and urban challenges to access.
May need to focus on other community priorities first.
Operationalize Institute of Medicine report recommendations regarding quality of cancer care
Work with NIH and professional societies to implement these recommendations.
Communicate across social science, clinical medicine, and basic research.
Prepare to respond and measure the imminent challenges Medicaid will face with the Affordable Care Act
Develop cancer quality metrics at the system level.
Discuss how to address health-care networks that do not accept Medicaid yet will serve large numbers of breast cancer patients at risk for inequities in care.
